# Vascularized Microfluidics and Their Untapped Potential for Discovery in Diseases of the Microvasculature

**DOI:** 10.1146/annurev-bioeng-091520-025358

**Published:** 2021-04-16

**Authors:** David R. Myers, Wilbur A. Lam

**Affiliations:** 1 The Wallace H. Coulter Department of Biomedical Engineering, Georgia Institute of Technology and Emory University, Atlanta, Georgia 30332, USA; 2 Department of Pediatrics, Division of Pediatric Hematology/Oncology, Aflac Cancer Center and Blood Disorders Service of Children’s Healthcare of Atlanta, Emory University School of Medicine, Atlanta, Georgia 30322, USA

**Keywords:** microvasculature, endothelial, microfluidics, blood, pathology

## Abstract

Microengineering advances have enabled the development of perfusable, endothelialized models of the microvasculature that recapitulate the unique biological and biophysical conditions of the microcirculation in vivo. Indeed, at that size scale (<100 μm)—where blood no longer behaves as a simple continuum fluid; blood cells approximate the size of the vessels themselves; and complex interactions among blood cells, plasma molecules, and the endothelium constantly ensue—vascularized microfluidics are ideal tools to investigate these microvascular phenomena. Moreover, perfusable, endothelialized microfluidics offer unique opportunities for investigating microvascular diseases by enabling systematic dissection of both the blood and vascular components of the pathophysiology at hand. We review (*a*) the state of the art in microvascular devices and (*b*) the myriad of microvascular diseases and pressing challenges. The engineering community has unique opportunities to innovate with new microvascular devices and to partner with biomedical researchers to usher in a new era of understanding and discovery of microvascular diseases.

## THE BIOLOGY, BIOPHYSICS, AND PATHOLOGY OF THE MICROVASCULATURE

### In the Microvasculature, Blood Cells Are on the Same Size Scale as Vessels

**Figure 1:**
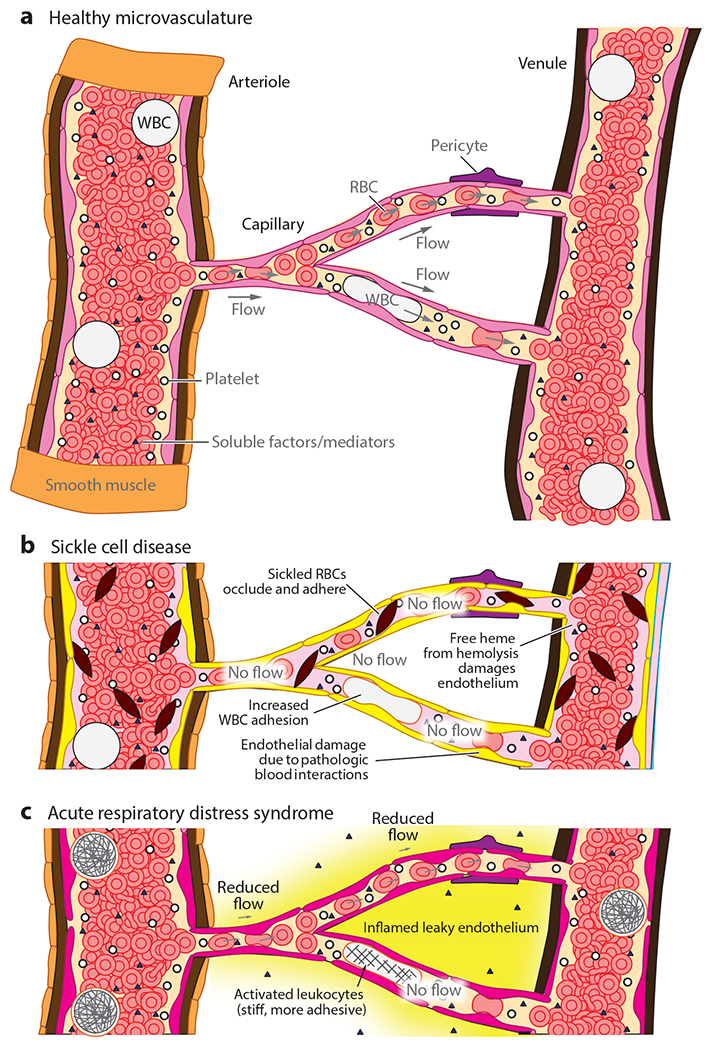
Diseases of the microvasculature can be caused by changes to the endothelium, blood, or their ensuing pathological interactions. (*a*) The microvasculature is composed of arterioles, capillaries, and venules. Blood cells must nominally deform to pass through the microvasculature, and changes to the adhesiveness or stiffness significantly influence transport. Soluble factors in blood can modulate endothelial cell, smooth muscle cell, and pericyte activity. (*b*) In sickle cell disease, sickled red blood cells (RBCs) are stiffer and occlude endothelium, white blood cells (WBCs) are more adhesive, and free heme damages the endothelium. (*c*) Acute respiratory distress syndrome involves an inflamed, leaky endothelium and activated leukocytes, which are stiff and more adhesive.

Microvascular diseases result from pathological interactions between blood cells and small vessels distributed throughout the body. As the vessels are on the same size scale as individual blood cells, blood can no longer be treated as a continuum, and single-cell biophysical events such as adhesion and altered deformability play a significant role in modulating the microvascular function (Figure 1a). For example, biophysically altered red cells in sickle cells are pathologically stiffened and adhesive, and those aberrant interactions with the microvascular endothelium cause up-regulation of endothelial inflammatory signals, leading to microvascular dysfunction and disease (Figure 1b). Similarly, in acute respiratory distress syndrome (ARDS), the inflammatory milieu activates leukocytes and renders them more adhesive and stiffened, causing obstruction of the pulmonary microvasculature, which leads to more inflammation (Figure 1c).

**Figure 2:**
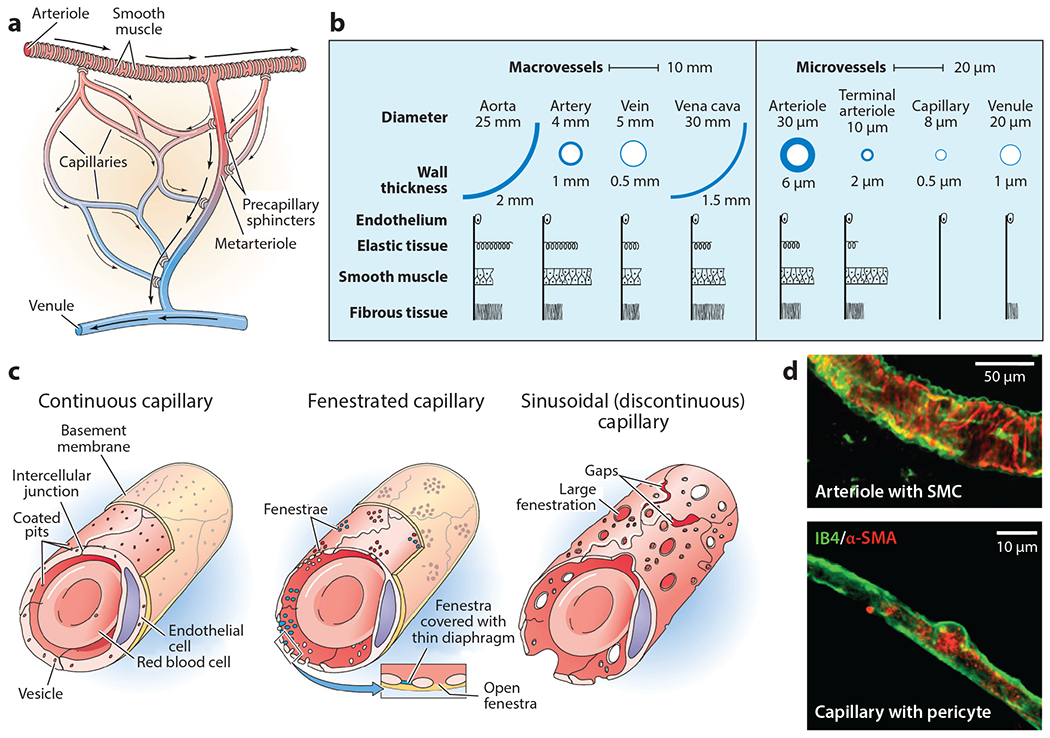
Anatomy and structure of the microvasculature. (*a*) The microvasculature includes valves that are present as precapillary sphincters and valves in the venules. Panel *a* adapted from Reference 116. (*b*) The microvascular vessels are defined as less than 100 μm thick and are much simpler in structure than arteries and veins. Panel *b* adapted from References 1 and 117. (*c*) Endothelial cells are highly heterogeneous, especially with regard to the anatomical source, which should be considered when designing an in vitro system. Panel *c* adapted from Reference 116. (*d*) Arterioles and capillaries are relatively simple structures with smooth muscle cells and pericytes, respectively Panel *d* adapted from Reference 39. Abbreviations: α-SMA, alpha smooth muscle actin; IB4, isolectin B4; SMC, smooth muscle cell.

The microvasculature vessels include arterioles, capillaries, and venules (1) and together constitute the vast majority of the cardiovascular system with a total length of 100,000 to 160,000 km (2) and an approximate surface area of 300 to 1,000 m^2^ (3). All the vessels are lined with endothelial cells, and the microvasculature contains approximately 98% of the endothelial cells in the cardiovascular system (4). Arterioles branch from arteries and have diameters ranging from 5 to 100 μm, and they contain a monolayer of endothelial cells, surrounded by one to two layers of smooth muscle, enclosed by a thin adventitial layer (5). The smooth muscle regulates flow by constriction or dilation. Capillaries branch from arterioles and have diameters of 5 to 10 μm, and they form an interconnecting network of tubes with an average length ranging from 0.5 to 1 mm. Capillaries only have a layer of endothelial cells and an associated basement membrane, and in some cases they are smaller than red blood cells (RBCs). Venules connect capillaries to veins and are approximately 20 μm in diameter; they are similar to arterioles, but lack smooth muscle (Figure 2).

Within the microvasculature, the endothelial cells, smooth muscle cells, and pericytes all play key roles in influencing function. The microvasculature influences numerous physiological and pathological processes including inflammation, healthy clotting (hemostasis), unhealthy clotting (thrombosis), angiogenesis, immunity via leukocyte trafficking, and selective transport of various nutrients, ions, and macromolecules (3, 4, 6). Endothelial cells play a key role in all of these functions and are highly heterogeneous in morphology and function depending on their anatomical location (3, 5) (Figure 2). Hence, when constructing in vitro mimics of the microvasculature, it is critically important to consider the origin and type of endothelial cell. In addition, endothelial cell behavior is modulated by soluble mediators, mechanical forces, cell-to-cell interactions with pericyte and smooth muscle cells, cell-to-matrix interactions, pH, and pO_2_ (3, 7), which can all vary in the microvasculature. The basal lamina is a key extracellular matrix structure bordering endothelial cells that provides structure and adhesion for endothelial cells while also influencing permeability, clotting, and cell migration. It contains glycoproteins; adhesion molecules such as laminin, fibronectins, entactin, and thrombospondin; various proteoglycans such as heparin sulfate that influence mechanics and hemostasis; and microfibril collagen types IV and V (8). Pericytes share a basement membrane and chemically and mechanically communicate with the endothelium (9), regulating capillary diameter, tight and adherens junctions, and extracellular matrix protein secretion (10). Higher pericyte density is associated with lower vascular permeability; for example, the pericyte-to-endothelial cell ratio is 1:1 in the brain, 1:10 in the skin and lungs, and 1:100 in smooth muscles (9). Although studies are still ongoing, evidence suggests that pericytes play key roles in regulating blood flow via capillary contraction, differentiate into other cell types including smooth muscle cells, and regulate capillary growth (7). Smooth muscle cells play a key role in regulating blood flow, signaling, and regulating vascular tone. In response to injury, the vasoconstriction response limits blood loss and helps reestablish hemostasis. Smooth muscle cells and pericytes communicate with endothelial cells via gap junctions and are thought to play an important role in specifying endothelial cell phenotypes. As with pericytes, the maturation of new vessels requires the recruitment of smooth muscle cells to reestablish vessel integrity (11).

Blood is primarily composed of RBCs, white blood cells, and platelets, within plasma containing numerous proteins. Nominally, the mechanical and biophysical properties of individual blood cells enable them to pass through the microvasculature. Canonically, RBCs carry oxygen and are anucleate disk-shaped cells with a thickness of 2 μm and a diameter of 8 μm. RBCs deform as they pass through the capillaries and nominally are highly deformable (5), measured to be ~200 Pa (12). White blood cells are a varied group of cells that play a central role in immunity and are larger cells with diameters ranging from 8 to 15 μm and a stiffness (for granulocytes) of ~350 Pa (12). White blood cells cross the endothelial barrier and also must deform to pass through capillaries. Platelets are small anucleate cells responsible for clotting that are capable of aggregating on exposed collagen to stem the wound and even applying muscle-like force on the order of 10 s to 100 nN (13, 14) to shrink and stiffen the clot. During clotting, damaged cells release tissue factor, which activates a cascade of proteases that amplify this signal, termed the coagulation cascade. One of the products of the coagulation cascade is thrombin, which activates platelets for clotting and polymerizes monomeric fibrinogen into polymeric fibrin. As blood regularly interacts with the endothelium, we refer the curious reader to our comprehensive review on the biophysics and mechanics of blood (15).

### Biophysics of the Microvasculature

At the microvasculature size scale, the biophysics of individual blood cells plays a significant role in microvasculature function and pathology. In vitro mimics of the microvasculature should be designed to biophysically imitate physiological microvasculature, including appropriate size, shear stress, viscosity, and microenvironmental stiffness. For convenience, this section provides references and quantitative numbers as a starting point to aid in the design of new systems. In many cases, data are limited due to the difficulty of performing in vivo measurements.

Microvascular vessels and their in vitro counterparts should have diameters that are on the same order of magnitude as individual blood cells. As such, blood cannot be treated as a simple continuum, and it is important to consider the stiffness and size of single blood cells. In the smallest capillaries, RBCs must deform to pass, so changes in microvasculature function and transport occur due to increases in RBC stiffness or adhesion, which is common in many cases, including sickle cell disease (16, 17), malaria (18, 19), or even various intravenous fluids (16). Adding to this complexity, the shear (20) and oxygen levels (21) in the local microenvironment also affect RBC adhesiveness. As in larger vessels, there is also evidence that stiffer cells (leukocytes, stiff RBCs) travel near the vessel’s periphery, suggesting that stiffer cells interact with the endothelium more than do their soft counterparts, although the implications of this remain to be seen. Finally, as cells travel through the microvasculature, they can locally change hydraulic resistance and chemotactic gradients (22).

Understanding and recapitulating the shear stress are critical, since many decades of research have demonstrated that shear stress heavily influences endothelial cell behavior and signaling both in vitro and in vivo (23, 24). In addition to morphologically changing and aligning with flow, shear stress influences signaling pathways related to growth factors [platelet-derived growth factor subunit B (PDGF-B), epidermal growth factor (EGF), etc.], coagulation [thrombomodulin, tissue plasminogen activator (tPA), tissue factor, protease activated receptor 1 (PAR-1), etc.], inflammation [nuclear factor kappa-light-chain-enhancer of activated B cells (NF-κB); vascular cell adhesion protein, intercellular adhesion molecule, platelet endothelial cell adhesion molecules (V/I/PE-CAMs); selectins, etc.], extracellular matrix degradation [collagen XII, matrix metallopeptidase 9 (MMP-9)], and others [cathepsin K, early growth response protein 1 (Egr-1), bone morphogenic protein 4 (BMP-4), etc.]. Fluidic shear stress also modulates genes responsible for cell division, differentiation, migration, and apoptosis, both in vitro and in vivo. Fluidic shear stress can also influence the permeability of the endothelium in vitro (6) and in vivo (23), which has a direct effect on the diffusion of substances across the endothelium.

Although limited, some data do provide quantitative values for shear stress in the microvasculature. Human conjunctival arterioles have shear rates ranging from 587 to 3,515 s^−1^ with wall shear stresses ranging from 1.7 to 21.1 Pa (1 dyn/cm^2^ = 0.1 Pa) (25). Shear stress in the conjunctival capillaries and venules is continuous and reaches a maximum of 9.5 Pa in the smallest capillaries and a minimum of 0.3 Pa in the largest diameter postcapillary venules (26). In rat glomerular capillaries, shear rates of 680–1,070 s^−1^ were found with shear stresses on the order of 3 Pa (27).

**Figure 3:**
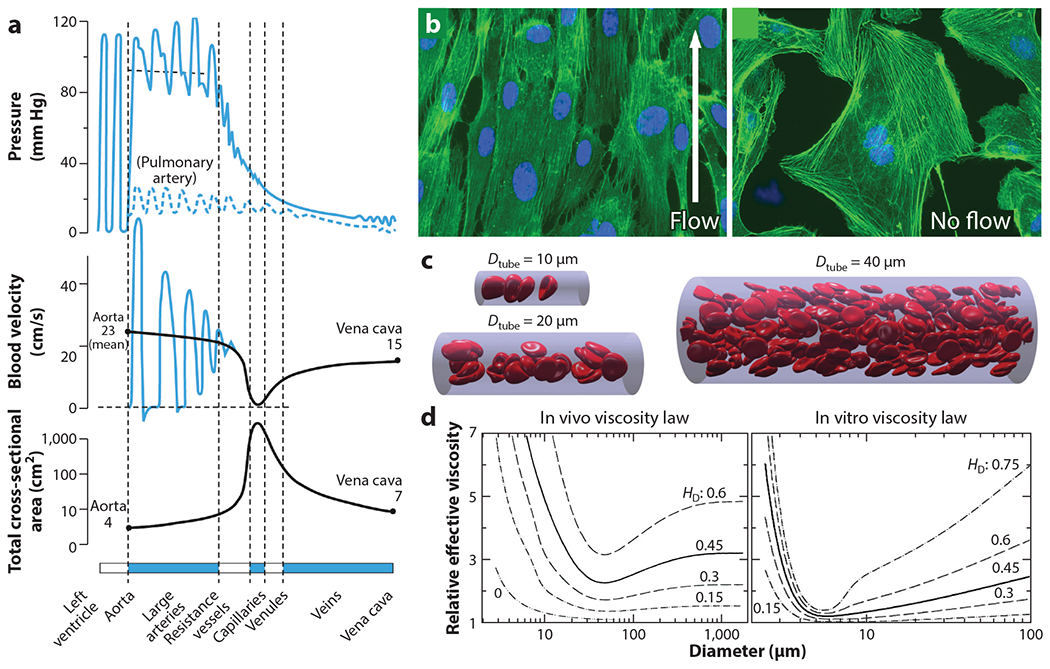
Biofluid mechanics of the microvasculature. (*a*) As blood transitions from the arteries to the arterioles, capillaries, and venules, there is a significant drop in the applied pressure and blood velocity due to a significant increase in the cross-sectional area of the vessels. Panel *a* adapted from Reference 1. (*b*) Endothelial cells strongly respond to shear stress, changing morphologically and with altered signaling pathways related to growth factors, coagulation, inflammation, extracellular matrix degradation, cell division, differentiation, migration, apoptosis, and permeability. Panel *b* adapted from Reference 118. (*c*) A low hematocrity (0.15) simulation illustrates the Fåhræus-Lindqvist effect and unique noncontinuum flow physics governing the microvasculature. Panel *c* adapted from Reference 119. (*d*) Mathematical models corrected with experimental data provide an estimate of the expected relative viscosity as a function of bulk hematocrit (H_D_). Notably, there can be up to a fourfold increase in smaller vessels in vivo, informed by rat mesentery microvascular measurements, as compared to in vitro, informed by glass capillary experiments. Empirically derived equations for both are available from Reference 32.

The size of the microvasculature, speed of fluid, and unique rheological properties of blood all play a role in influencing the shear stress. The microvasculature predominantly experiences laminar, continuous flow, with typical fluid velocities of 1 mm/s (Figure 3). Pulsatile flow can be induced by precapillary vessels, with flow ranging from zero to several millimeters per second (5). Blood itself is a shear thinning fluid, and this property largely arises from reversible RBC aggregation (<10 s^−1^) (28, 29) and, above 400 s^−1^, RBC rolling, tumbling, and, later, deformation (29). By extension, changing the volume percentage of RBCs in the blood, or hematocrit, also influences the viscosity (30). In all microvasculature vessels, the viscosity of blood decreases and has a dependence on the vessel diameter when the vessel diameter is smaller than 300 μm, as described by the Fåhræus-Lindqvist effect, in which a cell-free layer (~3 μm) occurs in vessels, reducing the effective viscosity via lower flow resistance (31) and leading to an apparent viscosity that is different from the bulk measurement. Interestingly, Fåhræus and Lindqvist (32) performed their measurements in glass capillaries. While the effective viscosity drops in vivo in small vessels, the magnitude of the drop was lower than predicted, especially in vessels with diameters <40 μm. This result was presumably from interactions between blood components and the inner vessel surface, such as endothelial-blood cell adhesion, an irregular inner vessel diameter, and tortuous vessel shape (32). Taken together, these results suggest that appropriate in vitro microvasculature models should seek to use the same size, fluid, and flow speeds as done in vivo to ensure a close approximation of the associated shear conditions.

Finally, it is critical not to overlook the importance of the microenvironmental stiffness and composition on microvasculature behavior. Endothelial cells, pericytes (33), and smooth muscle (34) all respond to microenvironmental stiffness in vitro and in vivo. For example, in vivo studies show that increased age-related stiffness of the murine thoracic aorta increases cell-cell junction width and permeability that are abrogated by inhibiting cell contractility. Presumably, this is consistent for a wide number of endothelial cells, including those of the microvasculature, although direct in vivo evidence has not been gathered. Designing in vitro systems to recapitulate the microvasculature stiffness can be difficult since direct data on the microvasculature stiffness are not available. However, it is reasonable to assume that the tissue stiffness (35) is between that of the softest tissues in the body at ~1 kPa (36) and that of the intimal tissue surrounding arteries, which has a stiffness of ~35 kPa (37). Notably, the endothelial cells themselves may locally influence stiffness since they actively deposit (35) and degrade extracellular matrix proteins (23, 24). Data suggest that both the ligand presentation of the basal lamina and stiffness synergize to influence cellular mechanotransduction (34).

### How Does Microvasculature Dysfunction Occur?

**Figure 4:**
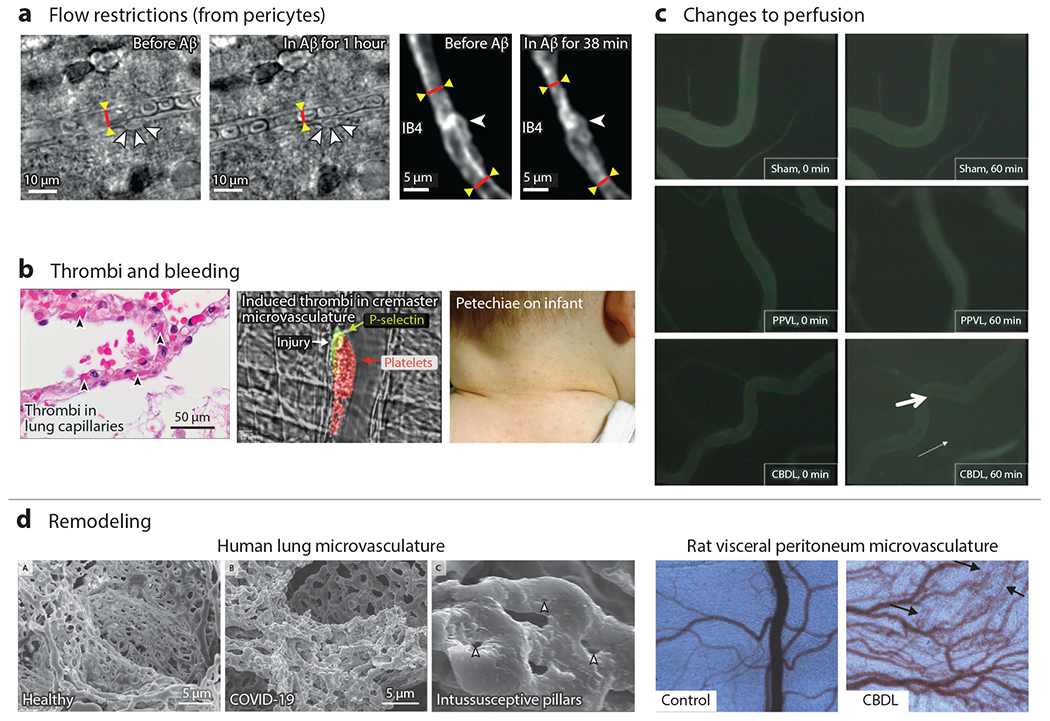
Microvascular dysfunction in vivo occurs from flow restrictions, thrombi, bleeding, microvascular remodeling, and changes to perfusion. (*a*) Bright-field and two-photon fluorescence of rat cortical slices show constriction near pericytes. Less conspicuous, albeit significant, systemic 25% changes in diameter can cause reductions in blood flow on the order of 50% in the brain (39). (*b*) Microthrombi, especially when systemically distributed, can cause organ damage and death. Here, microthrombi are shown from the alveolar capillaries of a patient who died from COVID-19 (*left*) or induced by laser injury in murine cremaster microvasculature (*middle*). Petechiae are from capillary bleeding, seen here on an infant with viral illness (*right*). Panel *b* adapted from References 38 (left subpanel), 46 (middle subpanel), and 120 (right subpanel). (*c*) Common bile duct ligation causes changes to the vascular permeability as seen by the increased fluorescent signal in the interstitium. Panel *c* adapted from Reference 121. (*d*) Microvasculature can also remodel in response to pathological conditions, shown here for a COVID-19 patient. In particular, intussusceptive angiogenesis appears to be occurring as evidenced by the intussusceptive pillars. Changes to the microvascular density in the visceral peritoneum of rats can be seen with common bile duct ligation. Panel *d* adapted from Reference 121. Abbreviations: Aβ, amyloid beta; CBDL, common bile duct ligation; IB4, isolectin B4; PPVL, partial portal vein ligation.

Microvasculature dysfunction manifests in several key ways, including altered blood flow, thrombosis aberrant bleeding, remodeling, and altered tissue permeability (Figure 4). Thrombosis, or aberrant clotting, occurs in a variety of disorders and may be the common pathological end point of problems, from increased endothelial adhesion to leukocytes or RBCs, to disseminated microclots or fibrin deposition (38). This resulting loss of blood flow to the area can lead to localized death and, if systemic, affect whole organs, and even lead to death. Thrombosis represents an extreme case of altered blood flow, but less conspicuous, albeit significant, changes may play a major role in pathology. For example, a systemic 25% pericyte-mediated reduction in capillary diameter in the brain is estimated to reduce blood flow by as much as 50% and contribute to a state of hypoxia (39). The microvasculature can also experience bleeding, which can be easily visualized in humans by the presence of petechiae, tiny purple, red, or brown spots on the skin created by blood that has leaked through skin capillaries. Various changes to the capillaries or blood contribute to this, including intuitive changes from low platelet counts, vessel inflammation (vasculitis), leukemia, loss of platelet function, and loss of clotting proteins. Nonintuitive changes can occur from bacterial and viral infections, such as the creation of cross-reactive antibodies that alter platelet function (40), as well as chemotherapy-induced cell stiffness changes that can obstruct capillaries (41).

Changes to endothelial cell permeability alter the transport of macromolecules, small molecules, and whole cells and have been tied to numerous hematological and inflammatory diseases such as sickle cell disease, malaria (35), and sepsis (42). Endothelial cell permeability can be influenced both by biochemical factors, such as cytokines and inflammatory mediators (42), and by biophysical factors, such as age-related intimal stiffening (37) and fluidic shear (43, 44). Increases in permeability are also the most common cause of edema where parenchymal and interstitial fluid accumulate, leading to changes in oxygen diffusion or pressure-related microvascular perfusion changes (42). The microvasculature may also remodel in response to pathological conditions through a variety of different processes, including angiogenesis, vasculogenesis, arteriogenesis, and intussusception. Numerous different conditions, including inflammation, ischemia, hypoxia, wound healing, aging, and mechanical force, contribute to this complex process and are covered in exquisite detail (45).

## PAST, PRESENT, AND NEW CHALLENGES FOR ENGINEERED IN VITRO MICROVASCULATURE

### Microfluidic Technologies Are Well Suited to Microvasculature Studies

Studying the microvasculature in vivo remains difficult. Imaging human microvessels in deep tissues is challenging due to optics limitations, although conjunctival imaging has been performed (26). Various animal models have been developed, including but not limited to live imaging the cremaster muscle arterioles in mice (46), excising and imaging arterioles from porcine hearts ex vivo (47), sacrificing an animal and measuring microvascular structure and flow resistance (48), and imaging capillary dynamics in hamster cheek pouches (1). Histological studies and functional measurements have helped identify key changes that occur in the microvasculature in various diseases. In addition, these models have played a key role in helping to elucidate biophysical parameters, such as order of magnitude estimates for flow speeds (26, 27). Some caution should be used, as many signaling pathways in individual blood cells and the endothelium can be significantly different from humans (49). In addition, even basic biophysical properties such as cell size can be significantly different (e.g., murine versus human RBCs). Understanding whether microvascular dysfunction causes or correlates with pathological conditions can also be difficult, as there is little control over the in vivo microvascular microenvironment. Given these limitations, in vitro mimics of the microvasculature could play a key role in elucidating the mechanisms of microvascular dysfunction in various pathological conditions. Importantly, a combined approach using both in vitro and in vivo models could be used synergistically to improve research rigor.

Microfluidics and biofabrication techniques are conducive to making in vitro models of the microvasculature. First, these techniques are able to create vessel-like structures (<100 μm) that are the same size as the microvasculature. Second, microfabrication techniques can create hierarchical vessels of comparable complexity. Third, these techniques yield models where common end points of microvascular dysfunction may be easily measured. Fourth, microfluidics only use small amounts of precious patient or murine samples (50), which is critical to successful translation to biomedical and clinical settings. Finally, in vitro microfluidic systems offer the unique opportunity to independently control many factors, such as endothelial type, platelet number, coagulation proteins, hematocrit, soluble agonists, shear stress, and leukocyte fractions. This level of control enables a detailed mechanistic study in which the role of both the blood and vascular components of the pathophysiology can be systematically dissected.

**Figure 5:**
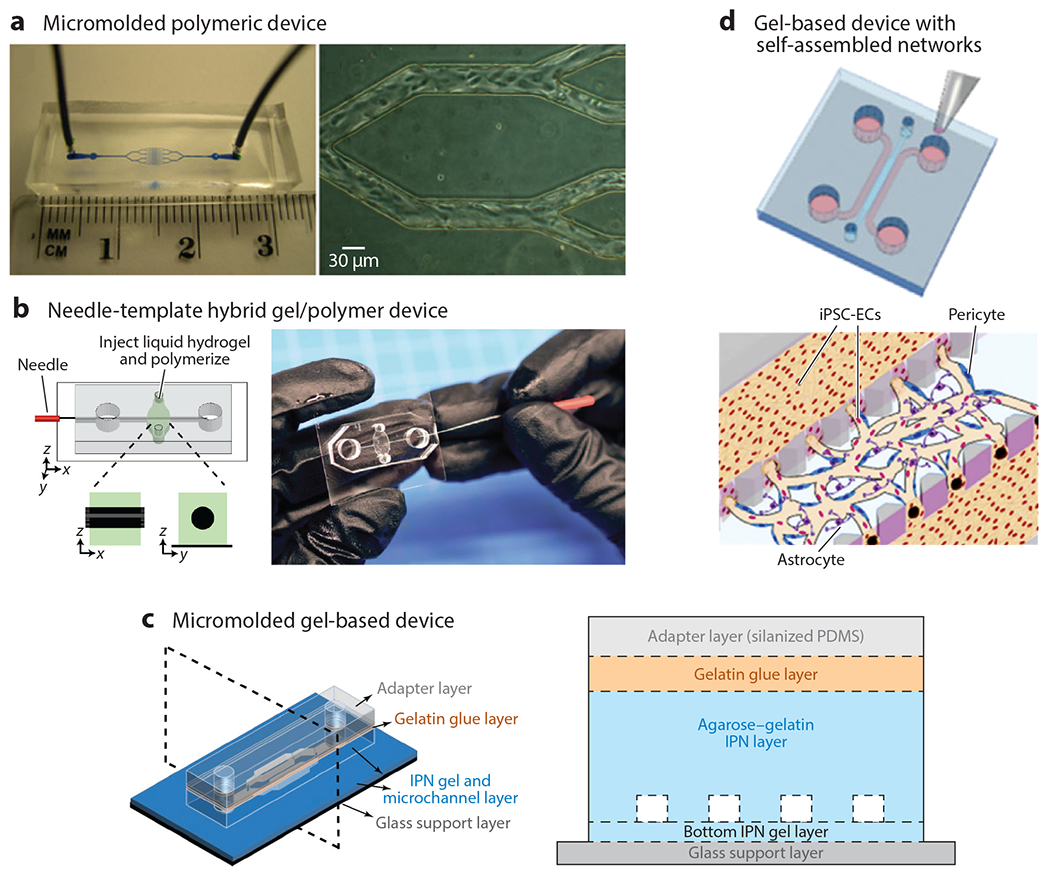
Approaches to creating in vitro microvasculature can be categorized as using polymeric or very soft gel materials with template-based, self-assembled, or directed networks of endothelial cells. (*a*) Polymeric-based micromolded structures offer high control over spatial dimensions and typically have a fast time to confluency. Panel *a* adapted from Reference 76. (*b*) Needle-templated hybrid structures offer key advantages of convenience and ease of fabrication, while using soft gels for perfusion studies, but typically are larger than most microvasculature (>100 μm). Panel *b* adapted from Reference 59. (*c*) Stiffer gels cleverly bonded with adapter layers led to the creation of the smallest in vitro vessels (~20 μm) that were still in a soft gel material for perfusion studies (35). (*d*) Pillars separating three fluidic channels enable gels laden with endothelial cells, pericytes, and astrocytes to be cast in the center gel and have perfusion in the adjacent channels to support culture and subsequent seeding after vascularization. Panel *d* adapted from Reference 75. Abbreviations: IPN, interpenetrating polymer network; iPSC-EC, induced pluripotent stem cell–derived endothelial cell; PDMS, polydimethylsiloxane.

Our review intends to complement the previously published excellent reviews on the use of microfluidics to study vascular function (51) and in vitro microvessels (9, 52, 53). One challenge to the novice is that the term microvessel can signify in vitro vessels that are hundreds of micrometers. At this size, these vessels do not mimic the biophysics of the microvasculature, which by definition has a diameter of less than 100 μm and is often much smaller. Hence, many excellent papers that focus on microvessel construction (>100 μm) are not covered here, as the primary focus is on technologies that meet the clinical definition of microvasculature (<100 μm). Broadly, two categories define the state-of-the-art in vitro microvasculature: the material choice (soft polymer, gel, or hybrid) and how the vessels are formed (template, directed growth, or self-assembled) (Figure 5).

Polydimethylsiloxane (PDMS) and soft polymer devices tend to be template based and excel at testing microfluidic obstruction and endothelial-cell interactions with a variety of endothelial cell types and blood samples from patients with various pathologies. Indeed, the first demonstration of an in vitro microvasculature model with patient blood was pioneered by our lab using this approach (54). Due to the ability to create complex mechanical structures and dense features, soft polymer template-based approaches have also led to advanced devices. For example, devices with four independent endothelialized channels in a microscopic viewing area enable the testing of four different conditions (16). Microfluidic resistance networks can be used to test three user-specified shear rates spanning two orders of magnitude in three sets of four endothelialized channels (55). Most recently, we integrated a valve to mimic wound creation and a model for bleeding (56), which complements existing approaches to studying hemostasis and thrombosis (57–58). There are several key disadvantages to these devices. First, the endothelial cells are mechanosensitive and attach to a hard matrix surface, which likely influences their function. Second, it is not possible to assess permeability in these devices, and permeability is a key metric of microvascular function. Finally, it is difficult to integrate other microvascular cell types such as pericytes into these systems.

Templates can also be used for soft gel–based devices. Template-based devices offer the advantage of highly controlled features, which enable high precision over fluidic flow conditions. In addition, by removing PDMS, endothelial cells are on a surface that is more physiological, and the permeability can be measured. The simplest templates available cast the gels around a needle (59, 60), glass capillary (61, 62), or metal wire (63). Although these tend to have large (>100 μm) features, these approaches played a significant role in advancing the field toward microvasculature-sized vessels, especially with the first demonstration of a sub-100-μm in vitro vessel (62). Indeed, subsequent work by the same group led to complex networks of sub-100-μm endothelialized vessels using micromolded gelatin cast in collagen, fibrin, or matrigel (64) and, later, the first vascularization of a capillary-sized channel (61). Subsequent work by other groups used similar micromolding approaches to create channels that were slightly larger than arterioles and venules at 120 × 150 μm and used these devices to study angiogenesis as well as hemostasis and thrombosis with blood (65, 66). Much smaller template-based soft-gel in vitro microvessels (30 μm) were achieved with agarose-gelatin interpenetrating polymer networks (35), which have a higher stiffness (5–50 kPa) than collagen and fibrin gels (10^−2^ to 10^0^ kPa) but are still substantially lower than typical polymeric devices (~1–3 MPa) (67). Pragmatically, this highlights how softer gels are more prone to collapse and can be more difficult to pattern with small features. An interesting approach to circumvent this challenge is to use light-based methods to photodegrade, ablate, or photopolymerize soft gels. Highly tortuous networks have been made using poly(ethylene glycol) diacrylate (PEGDA) hydrogels, with demonstrations of endothelialized channels on the order of 50 μm (68), and photoablation techniques have led to the first endothelialized capillary-sized vessel (69). An important yet often overlooked accomplishment of each of the aforementioned papers is the creation of a robust inorganic-organic interface between standard microfluidic tubing and the soft gels. Multiple approaches are taken but almost always involve a connection to PDMS or a housing to hold the soft device while still enabling microscopic visualization. A key drawback is that only endothelial cells have been integrated at this time, and no multicellular architectures have yet been realized. In addition, it can be more difficult to work with these systems given the fragility of the gel and the sometimes long culture periods of weeks. However, these systems have enormous potential, and the interested reader is directed to the excellent review in Reference 70.

Another approach to create in vitro microvascular networks relies on utilizing self-directed or directed growth approaches in soft materials such as fibrin gels or collagen gels (65) to facilitate the creation of networks that could test permeability and endothelial cell remodeling. Given the softness of these materials (~0.01–1 kPa), the gel is typically contained with a PDMS device or bonded to a carrier membrane of PDMS (35). Many methods take advantage of the increased viscosity and surface tension of the matrix materials and inject the gel between pillars (71) or similar structures (72–74) to trap it and enable subsequent fluidic access. Using this approach, in vitro microvessels can be self-assembled, by injecting endothelial cells with the gel, or directed, by applying endothelial cells to one side of the gel and setting up concentration gradients to encourage angiogenesis. Using template-based approaches provides a key advantage in that fully functional multicellular vessels on the size scale of venules (~30 μm) can be created, specifically with incorporated pericytes and astrocytes. Compared to their self-assembled endothelial-only counterparts, multicellular in vitro vessels are smaller, more strongly express tight junction proteins, and have reduced permeability (75). The central challenge with these self-assembled or directed growth approaches tends to be the heterogeneity in the vessel length, diameter, and networking, which complicate the interpretation of experimental data given the uncertainty in the fluid dynamics of the system. Understanding the exact fluid dynamics of these systems is a more complex endeavor, as precise methods to measure fluid flow at this scale are currently lacking and can be difficult to implement. At best, large-scale tracking of added particles or tagged cells may be used to estimate flow speeds, which could then be combined with assumptions about flow profiles and vessel shape to estimate shear stress.

### Ongoing Challenges and Design Considerations

After designing a new in vitro microvasculature or adapting an existing one based on size and flow characteristics, there are several logistical challenges that are worth considering in advance. First, air bubbles wreak havoc on any microfluidic system with cultured cells (76), but this can be remedied with proper technique (priming tubing and connecting fluidic ports in a bubble of liquid). Another consideration is the length of time until device maturity, which ranges from days in PDMS devices (54) to weeks in some gel devices (35), although a novel platelet-rich plasma-culturing approach may dramatically speed up this process (77, 78).

Related considerations are the length of time that these cultures will be exposed to blood and how the blood is anticoagulated, as each anticoagulant can introduce artifacts. Heparin prevents the generation of thrombin and would be difficult to use in studies related to aberrant bleeding and clotting. Ethylenediaminetetraacetic acid irreversibly chelates calcium while sodium citrate reversibly chelates calcium, which affects the coagulation cascade, platelet activation, endothelial cell function, endothelial adhesion, and leukocyte function. Using appropriate controls is a must (54). For clotting experiments with blood with calcium, corn trypsin inhibitor can be used since it inhibits Factor XII and keeps contact activation from damaged or charged surfaces from initiating clotting (56). Recalcifying blood in syringes leads to clots, although microfluidic mixers (79) may offer a path forward.

**Figure 6:**
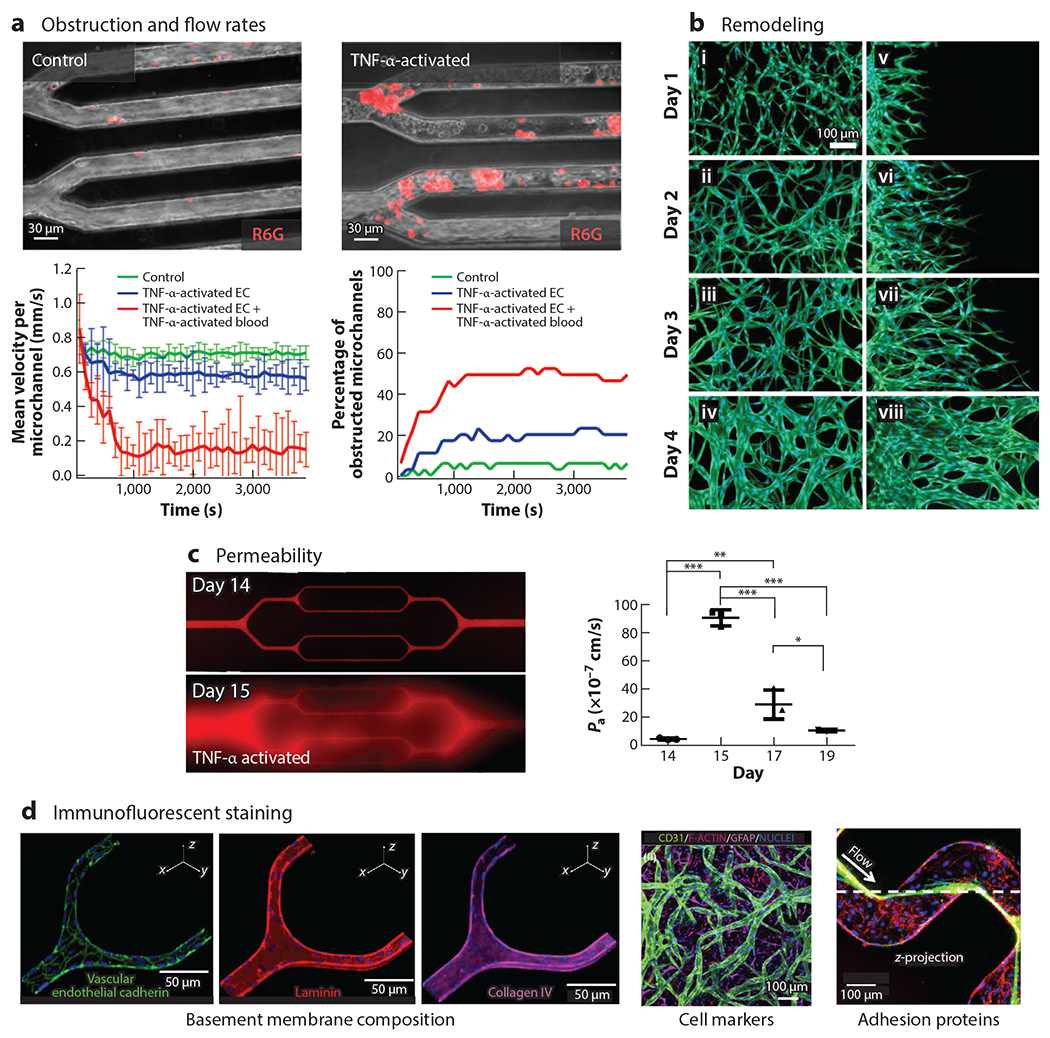
Measurements of in vitro microvasculature can measure all modes of dysfunction and help provide mechanistic data. As in vitro microvasculature is much easier to visualize than corresponding in vivo structures, it can provide a rich amount of quantitative information. (*a*) In vitro postcapillary venule obstruction and flow rates can be quantitated, here shown in response to TNF-α. Panel *a* adapted from Reference 54. (*b*) In vitro changes to microvasculature morphology and shape. Panel *b* adapted from Reference 71. (*c*) Exceptional permeability measurements can be performed and quantitated, especially ones lasting multiple days, to examine recovery of the microvasculature. Shown here is the permeability of the endothelium to BSA-AF549 tracer before and after TNF-α perfusion between days 14 and 15. *P* values were calculated using one-way analysis of variance with Bonferroni’s post hoc test (*P < 0.05, **P < 0.01, ***P < 0.001). Panel *c* adapted from Reference 35. (*d*) Immunofluorescent staining can be used to identify basement membrane composition, cell markers, and adhesion proteins, such as the von Willebrand factor, an adhesion that mediates platelet adhesion at high shear rates. Panel *d* adapted from References 35, 75, and 66, respectively. Abbreviations: EC, endothelial cell; GFAP, glial fibrillary acidic protein; TNF-α, tumor necrosis factor alpha.

The final key consideration for in vitro microvascular systems is determining which assays can reasonably be performed with these systems. Recalling that microvascular dysfunction can present as bleeding, thrombosis, remodeling, or altered perfusion, many of these functions can be directly visualized with microscopy (Figure 6). Immunofluorescent staining of key antigens and adhesion molecules can be done by simply perfusing microfluidics with appropriate buffers and primary/secondary antibodies. More traditional assays related to proteomics, lipidomics, and genomics require the pooling of multiple devices to attain appropriate numbers of cells for analysis, although newer single-cell-based techniques may obviate the need for pooled devices.

### Pressing Needs of the Field

With regards to engineering needs, one of the most pressing at the moment is for further miniaturization. Near capillary–sized in vitro vessels have only been created in a handful of situations (61, 69), most recently with a photoablation approach (69). While creating this size of microchannel poses some technical challenges, the larger challenge centers around the endothelialization of these channels given the relatively large size of detached endothelial cells as compared to the channel diameter (61, 69).

More research is also needed on developing protocols for culturing endothelial cells in fluidics, specifically the conditions needed to support endothelial cell viability. Given their widespread availability and well-established protocols, human umbilical vein endothelial cells are the predominant type of cell used for in vitro microvasculature, especially as they provide robust vessels. However, as endothelial cells are highly varied, more work is needed on using endothelial cells from other tissue sources. It can be difficult to culture these cells in microfluidics, and more work on appropriate protocols is warranted. In addition, it is important to better understand what processes control vessel size in assembled networks and to use this knowledge to develop fully functional capillary-sized vessels alone and hierarchical self-assembled networks that feature arteriole-, capillary-, and venule-sized vessels.

Another pressing need is the development of techniques capable of creating sub-100-μm microvessels that include both smooth muscles and endothelial cells. Important steps have been made in this direction, including multilayer cell assembly with fibronectin-gelatin glue (80), assembling stacks of cell-laden gel rings (81, 82), 3D stereolithography (83), and 3D bioprinting (84), although miniaturization is needed for all these approaches.

Numerous opportunities are available to further improve in vitro microvascular models with advanced engineering techniques, especially by providing better control over the local microenvironment. For example, microfluidics can be used to locally control the oxygenation levels in microvascular-sized structures (85), and are reviewed in detail in References 86 and 87, yet none to date have provided this level around an in vitro microvessel. Similarly, the microvasculature has venular valves (88) and precapillary sphincters (5) that likely influence function but have never been recapitulated and are difficult to study in vivo.

## MINDING THE GAP: BRIDGING ENGINEERING AND MICROVASCULAR RESEARCH

In vitro microvascular systems have significant potential in three main areas, as highlighted in the excellent review in Reference 89: therapeutic vascularization of damaged tissue, vascularizing engineered tissues to create better implants (90), and microphysiological systems. In vitro microvascular systems that do not use blood have made enormous contributions to our understanding of numerous topics, including metastasis (91), angiogenesis (89), improved drug delivery approaches (35), and the underpinning mechanisms and signaling pathways involved in barrier function (60).

The new frontier, and the focus of our review, is using in vitro microvascular systems with patient blood and/or patient-derived endothelial cells. These tools could play a key role in elucidating how pathological changes to endothelial cells and blood cells mechanistically lead to microvascular dysfunction (bleeding, clotting, permeability changes, and remodeling). Here, we provide an overview of the most significant microvasculature-related disorders and highlight pressing needs. In most of these diseases, little, if any, work has been done with in vitro microvascular systems. Our goal is to motivate progress in this area by pointing out numerous opportunities for areas of study and collaboration in both device development and clinical research. Working together, engineers partnering with clinicians, hematologists, and biologists have a unique opportunity to make significant headway in improving our understanding of many pathological conditions.

### Coronary Microvascular Disease

Cardiovascular disease remains one of the major causes of mortality worldwide and leads to atherothrombotic plaque formation and rupture, which, in turn, cause myocardial infarction. In addition to large vessels, several decades of research have shown that cardiovascular disease affects the microvasculature of the heart, which is now termed coronary microvascular disease (CMD). In CMD, which is also known as coronary small artery disease, coronary small vessel disease, or cardiac syndrome X, the diseased vessels are not associated with atherosclerotic plaques but do exhibit endothelial and smooth muscle dysfunction leading to vascular spasms and decreased blood flow. Because the coronary microcirculation regulates coronary blood flow in response to cardiac oxygen requirements, impairment of this mechanism, as in CMD, increases the risk of adverse cardiovascular clinical outcomes (92). In fact, CMD accounts for approximately two-thirds of clinical conditions presenting with symptoms and signs of myocardial ischemia without obstructive coronary disease in large vessels and even a small proportion of myocardial infarctions in which there is no obstructive large vessel coronary artery disease (93). Clinically, CMD occurs more in women and in individuals with diabetes, high blood pressure, or family history of cardiomyopathy. Pathophysiologically, CMD involves microvascular remodeling in the prearterioles, arterioles, and capillaries of the cardiac microvasculature that leads to microvascular obstruction. Remodeling is consistently linked to risk factors such as diabetes and hypertension and involves capillary rarefaction and narrowing of capillaries and intramural arterioles. Notably, some of these risk factors is also associated with modified blood cell biophysics, such as increased RBC stiffness and adhesiveness in diabetes (94). This stiffness and adhesion change exacerbates microvascular obstruction, although much remains to be learned about blood cell biophysics in the context of CMD. Treating CMD remains difficult, as the vasodilator response to pharmacological and physiological interventions is attenuated. Evidence suggests multiple causes, including endothelial dysfunction in the form of reduced vasoactive substances, such as nitric oxide, and smooth muscle cell thickening and attenuated response to vasodilators (92). Here, a vascularized model with integrated smooth muscle cells, endothelial cells, and blood, especially one capable of remodeling, could be a tremendous tool in revealing more detailed mechanistic insights. Table 1 lists key elements needed to model microvascular diseases with microfluidic systems.

### Cerebral Small Vessel Disease

Cerebral small vessel disease bears pathophysiologic similarities with CMD and in many ways is a catchall term indicating atherosclerotic changes in the cerebral microvasculature. One particular subset of cerebral small vessel disease that is of interest from a microfluidic modeling perspective is that of cerebral amyloid angiopathy (CAA). CAA is an important cause of intracerebral hemorrhage in the elderly and can occur as a sporadic disorder, in association with Alzheimer’s disease, or rarely as a genetic syndrome (95). In the nongenetic cause of CAA, amyloid peptides are initially deposited in the tunica media and adventitia and eventually accumulate in all layers of the vessel wall and cause loss of smooth muscle cells (96). These peptides are biochemically similar to the amyloid deposit comprising senile plaques in Alzheimer’s disease, the primary constituent of which is the amyloid beta peptide, a peptide fragment of the amyloid precursor protein. A recent paper examined the effects of amyloid plaques generated in Alzheimer’s disease and demonstrated that these molecules were capable of inducing pericyte constriction, which lowered cerebral blood flow and presumably contributed to cognitive issues (39). Of note is that CAA is strongly associated with atrial fibrillation of the heart, in which the arrhythmia leads to stasis and clotting of blood in the atria, which can then embolize in the cerebral microvasculature leading to stroke. While anticoagulant medications (e.g., warfarin, apixaban, rivaroxaban) are given to atrial fibrillation patients to prevent stroke, risk for hemorrhage is a known side effect of these medications. As such, because CAA patients often also suffer from atrial fibrillation, clinicians must balance the risk of stroke in atrial fibrillation, which can be prevented with anticoagulation, with the risk of hemorrhage in CAA, which is exacerbated by anticoagulation, with evidence-based guidelines or guidance from scientific data. A vascularized model of CAA, especially one that includes protein deposition, may provide insight into how to address this clinical conundrum (Table 1).

### Diabetic Microvascular Disease

Diabetes mellitus refers to several diseases that involve the dysregulation of insulin and glucose levels that results in hyperglycemia, or elevated blood glucose levels. Both type 1 and type 2 diabetes adversely affect the microvasculature in multiple organs, resulting in the classic, unique triad of retinopathy in the eye, nephropathy in the kidney, and neuropathy in the peripheral nerves. However, the microvasculature of skin, brain, adipose tissue, and cardiac and skeletal muscle is also affected in diabetes (97). While chronic hyperglycemia drives microvascular disease in type 1 and type 2 diabetes, hyperglycemia alone is not sufficient to trigger generalized systemic diabetic microvascular disease. Our current understanding of the pathogenesis involves a combination of direct glucose-mediated oxidative stress and the formation of advanced glycation end products due to superoxide overproduction, which are all toxic to endothelial cells. These, in turn, induce proinflammatory pathways in endothelial cells, which collectively result in altered blood flow and changes in endothelial permeability and coagulation resulting in organ dysfunction (98). Recent data demonstrate a relationship between blood pressure and progression of nephropathy and retinopathy, although the mechanisms remain unclear. Hence, the step-by-step underlying mechanisms of how other pathologic factors (e.g., blood pressure) affect and interact with elevated blood glucose levels to affect or accelerate pathophysiology remain unknown but provide ample opportunities for research. Due to the chronic nature of the disease process, vascularized microfluidic systems that enable endothelial cell culture over long timescales may be required, such as those developed by our laboratories (35) (Table 1).

### Sepsis

The combination of a microbial infection and dysregulated physiologic response leads to sepsis, a life-threatening infection with the presence of organ dysfunction. Sepsis represents a significant public burden, accounting for 5% of US health care costs in 2011 (99), and, in 2017, affecting 50 million people worldwide and accounting for 11 million deaths (100). Importantly, the pathobiology is still uncertain, and the syndrome is identified by several clinical signs and symptoms in patients with a suspected infection (99), including organ damage as evidenced by reductions in platelet counts, increases in bilirubin, increases in creatine, and reductions in partial oxygen pressure in breath. The immune system plays an essential role in mediating sepsis, and both proinflammatory and anti-inflammatory mediators (101) can lead to microvascular dysfunction due to damaged endothelial cells, neutrophil activation (102), changes to permeability, and aberrant clotting, as evidenced by inappropriate intravascular fibrin deposition (103). Considerable debate still exists on whether this coagulopathy contributes to the dysfunction or just occurs as a result of the syndrome (103) (Table 1).

### Disseminated Intravascular Coagulation

Disseminated intravascular coagulation (DIC) occurs when blood clots form throughout the body in small blood vessels, which can lead to life-threatening organ damage. Paradoxically, DIC can be accompanied by life-threatening bleeding, as the significant amount of clotting diminishes the circulating platelets and coagulation factors. DIC is typically caused by sepsis (30–50% of patients) as well as trauma and major surgery (45% of patients), but also arises from organ destruction, malignancy, and complications of pregnancy (104). Treating DIC remains exceptionally difficult and usually focuses on treating the underlying cause. While it is clear that there is a link between inflammation and coagulation, with extensive cross talk between these systems, much remains to be learned about the influence of the microvasculature and the biophysics of DIC. For example, while it is well established that mononuclear cells can express tissue factor, which leads to platelet activation and fibrin formation, little is known about how the microvascular microenvironment influences this process (Table 1). Separately, coronavirus disease 2019 (COVID-19) infections involve a coagulopathy that bears a significant resemblance to DIC, specifically microvascular thrombosis. However, there are some notable differences, such as a higher platelet count, higher coagulation factor levels, and only a mild drop in plasma levels of physiological anticoagulants in COVID-19. Unlike DIC, the coagulopathy is mostly prothrombotic, with fewer bleeding complications (105). Similar to DIC, in vitro microvasculature studies could play a key role in helping to elucidate the most significant factors creating disseminated clotting and may even find use as a therapeutic testing platform.

### Thrombotic Microangiopathies

Thrombotic microangiopathies are pathologies that result from microvasculature endothelial injury and include hemolytic uremic syndrome (HUS), atypical hemolytic uremic syndrome (aHUS), thrombotic thrombocytopenic purpura (TTP), and HELLP (hemolysis, elevated liver enzymes, and low platelet count). In all these pathologies, thrombosis occurs in the capillaries and arterioles due to endothelial cell injury. Schistocytes, or fragmented RBCs, are often associated with HUS and TTP (as well as DIC) and are thought to occur from an interaction with fibrin, although the exact biophysics of this event remains undefined. Pathologically, HUS and aHUS occur from endothelial cell damage, either by Shiga toxin producing *Escherichia coli* or complement activation. Endothelial cell damage also leads to leukocyte recruitment, increased thrombin generation, impaired fibrinolysis, and microvascular thrombosis. TTP occurs from a deficiency in ADAMTS13 (a disintegrin and metalloproteinase with a thrombospondin type 1 motif, member 13), which is responsible for cleaving very large multimers of von Willebrand factor. Without ADAMTS13, platelets aggregate on long strands of von Willebrand factor in the microvasculature, eventually leading to thrombi formation. In vitro microvasculature could help identify the pathogenesis of HELLP, which remains unclear and has been hypothesized to include changes to complement, hemostasis, ADAMTS13 activity, antiphospholipid antibodies, and inflammation (106) (Table 1).

### Sickle Cell Disease

Sickle cell disease (SCD) is a devastating monogenic disease in which a single point mutation results in abnormal hemoglobin molecules that polymerize into rigid fibers leading to RBC stiffening under acidic and hypoxic conditions, and, canonically, to increased blood viscosity and to the pathologic process of vaso-occlusion (107). Decades of research have qualitatively shown that alterations in the biophysical properties, such as cell deformability and cell adhesion, lead to interactions among sickle RBCs, platelets, reticulocytes (immature RBCs), white blood cells, and endothelial cells, and each of these likely contributes to microvascular occlusion, hemolysis, and endothelial dysfunction under different conditions—normoxic and hypoxic. Endothelialized microfluidics such as those developed by our laboratory (16, 54, 76) demonstrate that these systems are ideal for dissecting the myriad pathologic cellular and biomolecular biophysical interactions in this surprisingly complex disease. Ultimately, microvasculature-on-chip systems may help answer some of the biggest questions that plague clinicians who care for patients with SCD: What is the exact pathogenesis of the diffuse vasculopathy that pervades the entire body? Why are strokes, which are arterial and normoxic phenomena and are now known to occur as early as infancy, so prevalent in SCD patients? Why is there such variability in phenotype among patients who have the supposedly same genotype (Table 1)?

### Acute Respiratory Distress Syndrome

ARDS is a life-threatening form of respiratory failure characterized by widespread inflammation in the lung in which damage to the alveoli and microvasculature leads to significant lung edema (swelling), impaired gas exchange, and hypoxemia. The underlying causes of ARDS vary and can be either infectious or noninfectious in nature, including but not limited to pneumonia, sepsis, aspiration, shock, trauma, and inhalation injury. In the pulmonary vasculature, a cascade of inflammatory events propagates, including secretion of inflammatory cytokines such as TNF-α, IL-6, and IL-1β, activating the vascular endothelium and neutrophils. There, the activated neutrophils sequester and release additional biochemical inflammatory mediators, including oxidants, proteases, and neutrophil extracellular traps (108). In addition, as neutrophils are activated, actin polymerization and cell stiffening occur, leading to further neutrophil retention and clogging in the microcirculation (109) while endothelial permeability ensues. Despite a clear association between neutrophil influx into the lung microvasculature and disease severity, there is some debate as to whether neutrophils directly contribute to disease pathogenesis. Indeed, much of our data relies primarily on animal models, which may not translate to human pathophysiology. As such, ample opportunities remain for microvasculature-on-chip systems to resolve these issues (Table 1).

### Cancer

During tumor progression, a change in balance between angiogenesis stimulators and inhibitors and especially vascular endothelial growth factor induces capillary sprouts from the microvasculature (11, 110). These stimuli result in increased vascular permeability, accumulation of extravascular fibrin, and local proteolytic degradation of the basement membrane (7). Activated platelets and macrophages secrete growth factors, cytokines, proteases, and protease inhibitors that further influence this process. As vessels sprout, endothelial progenitor cells, cancer stem cells, and tumor cells can all be recruited to the new vessel, influencing the overall function (11). In addition to sprouting, the microvasculature also remodels via intussusception, although much less is known in comparison to angiogenesis (111). Intussusception involves the formation of interstitial columns in existing lumens that are invaded by periendothelial cells and expand to partition the lumen (Table 1).

### Psoriasis/Rheumatoid Arthritis

Psoriasis is known by the formation of thickened scaly plaques, itching, and inflammation, typically on the scalp, elbows, knees, and back. While characterized by an excessive proliferation of keratinocytes, the earliest detectable morphological change in psoriasis involves the dilation, increased permeability, and changes to the tortuosity of microvasculature in the papillary dermis as well as angiogenesis (112). It is thought that the larger microvasculature also facilitates T cell trafficking to maintain the plaque and provides increased blood flow (112). Rheumatoid arthritis is known by synovial inflammation and the occlusion of the microvasculature. The entire synovium becomes enlarged (hypertrophic) and heavily vascularized (hypervascular). Within the synovium, synoviocytes secrete increased levels of inflammatory and chemotactic factors, and significant population increases of lymphocytes, natural killer cells, synovial B cells, dendritic cells, and polymorphonuclear cells are observed (113) (Table 1).

### Vascular Malformations

Vascular formations are a collection of disorders (114) characterized by abnormal blood vessel growth; although present at birth, they become apparent at different ages. Canonically, they are thought to occur from arrested development of vessels at various stages of embryogenesis (115). They have the potential to grow and proliferate from internal stimuli such as menstruation, pregnancy, and hormonal release or external stimuli such as trauma or surgery. These formations can be associated with coagulopathy due to stasis of blood within the structures, which is known as localized intravascular coagulopathy. However, much remains to be learned about the origin and progression of these formations, especially in regard to the microvasculature.

## TEAMWORK MAKES THE DREAM WORK

In vitro microvasculature technologies have generated a significant amount of enthusiasm from many biomedical researchers as they can answer numerous questions in hematology, oncology, vessel biology, and infectious disease. Our hope is that this article serves as a springboard for new collaborations between biomedical researchers and engineers that ultimately lead to groundbreaking cures for many microvascular disorders.

## Figures and Tables

**Table 1:** Modeling diseases of microvascular dysfunction using vascularized microfluidics

	Characteristics of perfusate, blood, and/or blood cells	Vascular and endothelial cell type(s)	Affected organ(s)	Functional capabilities
Coronary microvascular disease	Depends on specific experiment	Coronary microvascular endothelial cells, smooth muscle cells	Heart	Vasospasm, endothelial dysfunction, microvascular obstruction, varied shear rates
Cerebral amyloid angiopathy	Deposition of amyloid beta on endothelial cells, inclusion of anticoagulant medications in perfusate/blood	Brain microvascular endothelial cells, smooth muscle cells	Brain	Vasospasm, microvascular obstruction, microvascular hemorrhage, varied shear rates, protein deposition
Diabetic microvascular disease	Elevated glucose in perfusate/blood, advanced glycation end products, inflammatory mediators (e.g., cytokines)	Microvascular endothelial cells of the target organ, smooth muscle cells (depending on type of experiment)	Classically, eye (retina), kidney, and peripheral nerves, but also skin, brain, adipose tissue, and cardiac and skeletal muscle	Endothelial dysfunction, vasospasm, thrombosis, varied shear rates
Sepsis and disseminated intravascular coagulation	Depending on severity: increased white blood cell count; decreased platelet count; elevated glucose, creatinine, liver enzymes, or serum lactate; decreased coagulation factors (longer clotting times)	Microvascular endothelial cells of the target organ, white blood cells (mononuclear cells)	Lung and kidneys, followed by brain, heart, liver, spleen, adrenal glands, pancreas, and gastrointestinal tract	Endothelial dysfunction, vasospasm, thrombosis, hemorrhage, varied shear rates
Thrombotic microangiopathies	HUS: Shiga toxin; aHUS: complement activation; TTP: ADAMTS13 deficiency; HELLP: unknown	Microvascular endothelial cells of the target organ (e.g., kidney glomerulus)	HUS: kidney; TTP: kidney, heart, brain; HELLP: liver	Endothelial dysfunction, vasospasm, thrombosis, hemorrhage, varied shear rates
Sickle cell disease	Blood or red blood cells from sickle cell disease patients or transgenic murine models, leukocytes, and platelets activated due to inflammatory conditions	Microvascular endothelial cells of the target organ (i.e., lung, kidney, brain)	All but lung, kidney, and brain are of specific interest	Endothelial dysfunction, thrombosis, in vitro sickle cell vaso-occlusion, varied shear rates, varied oxygen levels ranging from hypoxia to normoxia
Acute respiratory distress syndrome	Neutrophils activated with inflammatory mediators	Pulmonary microvascular endothelial cells	Lung	Endothelial dysfunction, thrombosis, neutrophil retention, and microvascular occlusion, varied shear rates
Cancer	Increased angiogenic factor stimulators; decreased inhibitors, vascular endothelial growth factor gradient, circulating cancer, and endothelial progenitors	Microvascular endothelial cells of the target organ, pericytes, platelets, macrophages, endothelial progenitor cells, tumor initiating cells, and more (11)	All, depending on cancer type and location	Endothelial remodeling, thrombosis, permeability, hypoxia, varied shear rates
Psoriasis/rheumatoid arthritis	Microvascular endothelial cells of the target organ, smooth muscle cells, specialized postcapillary venules, lymphocytes, polymorphonuclear cells	Psoriasis: angiogenic factors (112); rheumatoid arthritis: synoviocyte secreted IL-1, IL-6, TGF-β, IL-8, C5a, leukotriene B4 (122)	Papillary dermis (psoriasis), synovium (rheumatoid arthritis)	Endothelial remodeling, thrombosis, immune cell trafficking, varied shear rates

All vessels of the microvasculature (capillaries, prearterioles, arterioles, and venules) are implicated in these diseases. Abbreviations: aHUS, atypical hemolytic uremic syndrome; HELLP, hemolysis, elevated liver enzymes, and low platelet count; HUS, hemolytic uremic syndrome; TTP, thrombotic thrombocytopenic purpura.
